# Transactions of the Homœopathic Medical Society of the State of New York

**Published:** 1891-09

**Authors:** 


### Transactions of The Homœopathic Medical Society of the State of New York, for the Year 1890. Vol. xxv. Edited by the Secretary, John L. Moffat, M. D.

A bulky volume containing much of value and much of otherwise. A large portion is taken up with the report of the Committee on Medical Legislation, and it shows the committee to have been an active one. It did much work and accomplished its aim—that is, Homœopathy occupies as good a legal position in New York as does old-school medicine.

